# Participation Following Inpatient Rehabilitation for Traumatic Disorders of Consciousness: A TBI Model Systems Study

**DOI:** 10.3389/fneur.2019.01314

**Published:** 2019-12-18

**Authors:** Christopher Malone, Kimberly S. Erler, Joseph T. Giacino, Flora M. Hammond, Shannon B. Juengst, Joseph J. Locascio, Risa Nakase-Richardson, Monica Verduzco-Gutierrez, John Whyte, Nathan Zasler, Yelena G. Bodien

**Affiliations:** ^1^Department of Physical Medicine and Rehabilitation, Harvard Medical School, Boston, MA, United States; ^2^Neurorehabilitation Laboratory, Spaulding Rehabilitation Hospital, Boston, MA, United States; ^3^Department of Occupational Therapy, MGH Institute of Health Professions, Boston, MA, United States; ^4^Department of Physical Medicine and Rehabilitation, Indiana University School of Medicine, Indianapolis, IN, United States; ^5^Department of Physical Medicine and Rehabilitation, University of Texas Southwestern Medical Center, Dallas, TX, United States; ^6^Department of Neurology, Massachusetts General Hospital, Boston, MA, United States; ^7^Mental Health and Behavioral Science, Defense and Veterans Brain Injury Center, James A. Haley Veterans Hospital, Tampa, FL, United States; ^8^Division of Pulmonary and Sleep Medicine, Department of Internal Medicine, University of South Florida, Tampa, FL, United States; ^9^Department of Physical Medicine and Rehabilitation, McGovern Medical School at the University of Texas Health Science Center at Houston, and TIRR Memorial Hermann, Houston, TX, United States; ^10^Moss Rehabilitation Research Institute, Elkins Park, PA, United States; ^11^Department of Physical Medicine and Rehabilitation, Concussion Care Centre of Virginia, Ltd. and Tree of Life Services, Virginia Commonwealth University, Richmond, VA, United States

**Keywords:** traumatic brain injury, minimally conscious state, participation, rehabilitation, outcome

## Abstract

Severe traumatic brain injury (TBI) may result in a disorder of consciousness (DoC) and lead to substantial long-term disability. While level of independence with activities of daily living, especially for persons who recover consciousness during inpatient rehabilitation, generally improves over time, the degree of change in participation remains unknown. We determined level of participation among persons with TBI between 2005 and 2017 who were admitted to inpatient rehabilitation unable to follow commands and subsequently enrolled in the TBI Model Systems National Database. Participation on the Participation Assessment with Recombined Tools-Objective (PART-O) Productivity, Social Relations, and Out and About subscales was evaluated at 1–5 years post-injury. We used a mixed-effects model to longitudinally compare participation between persons who did and did not regain command-following during inpatient rehabilitation. We further explored the level of participation associated with increasing levels of functional independence (FIM). The analysis included 333 persons (229 recovered command-following during rehabilitation, mean age = 35.46 years, 74.9% male). Participation across groups, at all follow-up time points, on all PART-O subscales, was remarkably low (mean range = 0.021–1.91, maximum possible score = 5). Performance was highest on the Social Relations subscale and lowest on the Productivity subscale. Longitudinal analyses revealed no difference in level of participation or change in participation across time for persons who regained command-following during rehabilitation compared to those who did not. While productivity increased over time, social participation did not and participation outside the home increased more for younger than for older persons. Across all three PART-O subscales, FIM Motor scores positively predicted participation. FIM Cognitive scores positively predicted level of participation on the Productivity and Social Relations subscales. Exploratory analyses revealed that even persons who achieved independence on the FIM Motor and Cognitive subscales had low levels of participation across domains and follow-up years. In summary, persons with severe TBI who were admitted to inpatient rehabilitation unable to follow commands were found to be unlikely to participate in productive tasks, social endeavors, or activities outside of the home up to 5 years post-injury, even if functional independence was recovered.

## Introduction

Medical advances have improved the ability of healthcare providers to prevent early death among persons with severe traumatic brain injury (TBI) (1). Surviving persons may experience a disorder of consciousness (DoC), which includes the vegetative state, characterized by periods of eye-opening but no behavioral evidence of conscious awareness, and minimally conscious state, characterized by clearly-discernible but inconsistent behavioral signs of conscious awareness (2). Recovery of command-following (e.g., the ability to accurately respond to a spoken or written prompt such as “look up,” “make a fist,” “kick your leg”) is a critical clinical milestone in the recovery from a DoC, as it demonstrates increased situational awareness (3). For persons admitted to inpatient rehabilitation without command-following, restoration of independence across domains of self-care, mobility, and to a lesser extent, cognition, is possible for up to 10 years post-injury, especially for those who regain command-following during rehabilitation (4–6). However, less is known about whether these individuals eventually participate in social and productive activities. In fact, many studies of participation after TBI exclude persons with the most severe impairments and lower levels of function for logistical reasons (e.g., inability to complete self-report questionnaires or primary residence in a non-community setting) (7, 8). Alternatively, patients with severe TBI and prolonged impairments may be grouped with less severe patient populations (e.g., moderate TBI) (9), thus potentially masking cohort-specific effects (10, 11). Prior studies of severe TBI have focused on emergence from a DoC, global function, or recovery of basic cognitive abilities as primary outcome metrics; however, participation in community-based activities is also considered a measure of successful recovery after TBI. A more comprehensive understanding of participation across the trajectory of recovery from traumatic DoC may provide targets for early interventions and opportunities for instrument development, as well as inform programmatic changes that meet the unique needs of this population.

Although a precise operational definition of participation has not emerged from the literature, it is widely recognized to encompass varied life domains at the societal level, such as home activities, learning, social interactions (12–14), and productivity (15, 16). The World Health Organization International Classification of Functioning, Disability and Health (ICF) defines participation as “involvement in a life situation” and conceptualizes participation as one of the major components of function and disability in the context of health (17). Although return to effective functioning in the home, work, and social environments is a primary goal of rehabilitation after TBI, long-term improvements in these domains may not always occur (18).

Inability to return to work, attend school, or engage in other valued roles or routines, including those associated with leisure, is common during the years after moderate to severe TBI (11, 19–22). The few studies that include individuals with delayed, or no, recovery of command-following after TBI suggest that impaired participation is a predominant source of long-term societal burden (20). Furthermore, decreased participation across domains is not simply due to reallocating cognitive and psychological resources toward alternative activities (e.g., decreasing leisure to engage in more work activities) (11, 19). While a small number of demographic and injury characteristics have been found to predict participation (e.g., younger age at injury, higher levels of motor function at rehabilitation discharge) (23, 24), personal (e.g., social support) and psychological factors (e.g., psychological resilience) have the strongest influence (25).

Despite improved knowledge of long-term clinical outcomes, participation among individuals who have experienced very severe TBI remains understudied. A more complete understanding of participation over time is necessary to develop or refine participation measures, inform interventions to improve participation, and potentially reduce monetary and emotional burden. This study aims to: (1) characterize the level of participation across the domains of productive, social, and outside activities at 1, 2, and 5 years post injury among persons admitted to acute inpatient rehabilitation without command-following; and (2) compare longitudinal levels of participation between persons who regained and those who did not regain command-following during inpatient rehabilitation. We hypothesized that persons who regained command-following during inpatient rehabilitation would demonstrate higher levels of participation compared to those who did not regain command-following during inpatient rehabilitation. We also explored the relationship between functional recovery and participation to determine whether individuals who regain independence become productive, socially integrated and able to participate in activities outside the home.

## Materials and Methods

### Participants

**Figure 1:**
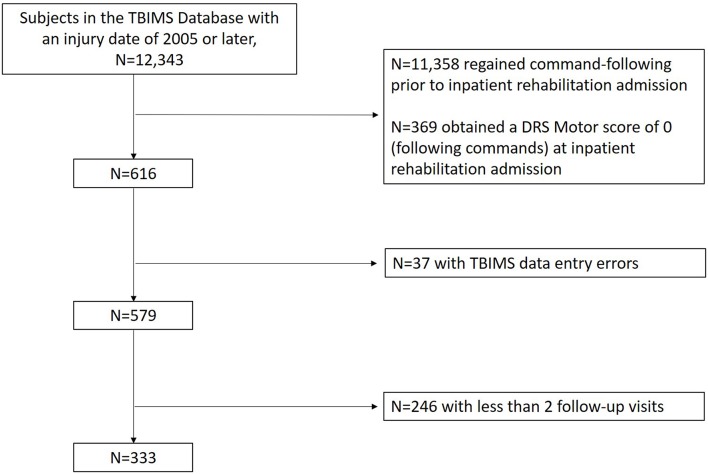
Participant Flow Diagram.

The sample was drawn from the Traumatic Brain Injury Model Systems (TBIMS) National Database (NDB). The TBIMS NDB is a longitudinal multicenter study which prospectively enrolls and collects data from individuals with moderate to severe TBI hospitalized and later admitted to inpatient rehabilitation facilities in the United States. The TBIMS is currently comprised of 16 regionally and demographically diverse centers. Each center obtains approval to contribute data to the database from their local Institutional Review Board (IRB) and obtains consent from persons with TBI or surrogates as per IRB protocol. A model system must include a Level 1 Trauma Center, acute neurosurgical care, comprehensive inpatient rehabilitation services, and multi-disciplinary rehabilitation and follow-up care. TBI was considered moderate to severe if there was documented evidence of post traumatic amnesia for greater than a day, loss of consciousness >30 min, a Glasgow Coma Scale (GCS) (26) score <13 in the Emergency Department, or intracranial neuroimaging abnormalities. An individual must meet the following criteria to be enrolled in the TBIMS NDB: be at least 16 years old, received care in a TBIMS center within 72 h of injury, and transferred directly from acute care to an affiliated inpatient rehabilitation program. For each participant enrolled in the TBIMS NDB, medical charts were reviewed and an in-person interview, with either the patient or a surrogate, was conducted to collect data on demographics, injury characteristics, and premorbid medical history. Follow-up interviews focusing on recovery of function were conducted at 1, 2, and 5 years post-injury with the participant or surrogate. A data quality review from the TBIMS NDB revealed systematic data entry errors at a single data collection site for variables related to determining command-following and this center was excluded from analysis (37 potential participants excluded). We included all other TBIMS NDB participants whose injury occurred in 2005 or later, who had not regained command-following by the time of their inpatient rehabilitation admission and had subscale scores from the Participation Assessment with Recombined Tools-Objective (PART-O) completed for at least two follow-up visits (at 1, 2, or 5 years post-injury) (Figure 1).

The primary analysis divided the sample into those who regained command-following during inpatient rehabilitation [Rehab Command-following (RCF)] vs. those that did not [No Rehab Command-following (nRCF)]. In addition, we conducted a secondary analysis (available in Supplementary Digital Content) that divided the cohort of individuals admitted to inpatient rehabilitation without command-following into those who did and did not regain command-following within 28 days post injury (TFC28, nTFC28, respectively) as this timeframe represents the accepted definition of “prolonged” DoC (6).

### Instruments

At the time of participant or proxy consent for TBIMS NDB enrollment, acute care charts were reviewed for demographic and injury characteristics (e.g., age at injury, sex, race, living settings, comorbidities, years of education, time to follow commands, etc.). Additionally, the results of the following assessments were obtained from the TBIMS NDB.

#### Command-Following

The presence of command-following was determined using two TBIMS variables at the time of rehabilitation admission, consistent with prior TBIMS DoC studies (4, 6). The first variable was the date a participant exhibited command-following on two consecutive assessments within a 24-h period following TBI. This was determined by reviewing acute rehabilitation medical records for documented evidence of command-following on the Glasgow Coma Scale (GCS, command-following is indicated by a motor subscale score of 6) (26). The second variable was the Disability Rating Scale (DRS) motor score (which includes the same items as the GCS motor score, but is inversely scored such that a DRS score of 0 corresponds to command-following) collected at rehabilitation admission (27). Participants were included in the cohort if both variables indicated no evidence of command-following prior to or at the time of inpatient rehabilitation admission.

#### Participation

The Participation Assessment with Recombined Tools-Objective The PART-O was the main outcome measure of participation. The PART-O is a 17-item self-reported or proxy-reported questionnaire developed specifically for use in persons with TBI. It is administered at every follow-up data collection point for the TBIMS NDB (28, 29). The PART-O provides estimates of participation across three life domains: (1) Productivity—time spent working, at school or on homemaking activities; (2) Social Relations—time spent with friends, giving emotional support, and internet communication; and (3) Out and About—days spent outside the home for leisure, shopping, or other purposes. Items within each domain are averaged to achieve a subscale score that can range from 0 to 5, with higher scores signifying greater levels of participation (29). Subscale scores may be derived when more than 50% of the items are answered. The PART-O has reasonable psychometric properties of person (0.86) and item (0.99) reliability (28). An alternative Rasch-adjusted scoring method was also developed to convert the measure to a unidimensional scale (30). However, because the unidimensionality of participation is not clear (31), and participation may manifest differently for individuals with a DoC (14), the present study utilized scoring based on the three separate subscales.

#### Functional Status

The FIM is an 18-item standardized measure of self-care, mobility, and cognition, specifically examining level of assistance required in basic activities of daily living (32, 33). It was scored by clinical providers during inpatient rehabilitation and obtained via telephone interview with the participant or proxy in later follow-ups. Scores are summed into a cognitive subscale (range: 5–35), a motor subscale (range: 13–91), and a total FIM score (range: 18–126), with higher scores representing a greater degree of independence.

#### Data Analysis

Demographic data for the early and late recovery groups were analyzed separately. A Shapiro-Wilk test revealed that age, years of education, and injury severity measures (i.e., GCS at admission, length of treatment in acute and rehab, and DRS and FIM scores at admission and discharge from rehab) were not normally distributed (*p* < 0.001). Therefore, these variables are reported as medians and interquartile ranges. Continuous demographic data were compared statistically using Mann-Whitney *U* test. Racial and gender compositions were compared using chi-squared tests of association. Cross-sectional analyses comparing the two rehabilitation groups on subscale PART-O scores (i.e., Productivity, Social Relations, Out and About) at each follow-up year were conducted with univariate analysis of covariance with the covariates of age, FIM Motor, and FIM Cognitive scores at time of follow-up. We also explored the relationship of functional status and participation at each follow-up where FIM Motor and Cognitive subscale scores were recoded into ordinal variables with three levels of performance. To better understand the relationship between functional independence and participation, we binned FIM Motor and Cognitive subscale scores into three levels and evaluated median PART-O scores at each FIM bin across subscales and follow-up years. The three FIM bins are based on categories of independence that reflect the underlying individual item rating definitions: Total Assistance Needed (Bin 1, Motor FIM scores = 18–38, Cognitive FIM scores = 5–14), Minimal/Moderate Assistance (Bin 2, Motor FIM scores = 39–77, Cognitive FIM scores = 15–29), and Complete Independence (Bin 3, Motor FIM scores = 78–91, Cognitive FIM scores = 30–35).

Longitudinal analyses were conducted as separate mixed-effects backward elimination models (*p* < 0.05 cutoff) for each of the three PART-O subscales (Productivity, Social Relations, and Out and About) as dependent variables. All three models began with the same fixed predictors and variances/covariances of random terms. Fixed terms consisted of our primary variable of interest, Command-Follow group (command-following recovered during rehabilitation [RCF] vs. command-following not recovered during rehabilitation [nRCF]), as well as year of follow-up (linear and quadratic function), age at injury, gender, length of stay in inpatient rehabilitation, and length of stay in acute care facility. Scores on the FIM Motor and FIM Cognitive subscales were included as time-varying covariates. Two-way interactions of group by year of follow-up, group by age at injury, and age at injury by year of follow-up as well as a three-way interaction of group by age at injury by year of follow-up were also included. The initial random terms were subject intercept, subject's linear term for year of follow-up, and their correlation. If the interaction between a fixed predictor and year of follow-up was significant, that would indicate that the linear trajectory of participation varied with the predictor variable. If a predictor term did not have a significant interaction with year of follow-up, but did have a significant main effect, then that would indicate that the predictor had the same influence on participation regardless of when it was measured, but that it did not predict the trajectory of participation over time. The backward elimination approach was a limited one intended primarily to pretest and remove, if non-significant, higher order interactions, quadratic terms, covariate terms, and random effects. Non-significant terms with the highest *p* value were systematically removed in a stepwise manner until only significant terms remained. By convention, non-significant lower order terms subsumed within significant higher order terms are permitted to remain in the model. Percent of dependent variable variance accounted for by fixed and random predictor terms were computed. Model residuals from fixed and random term predicted values were checked graphically for adherence to assumptions of normality and homoscedasticity, and to assess model fit. Only participants with at least two time points with non-missing dependent variable scores qualified for inclusion in any of the longitudinal analyses.

For the secondary analysis, we repeated the longitudinal analyses with the groups defined by those who did and did not regain command-following within 28 days post injury (TFC28, nTFC28 respectively) rather than those who did and did not regain command-following during inpatient rehabilitation. Analyses were conducted using SAS, version 9.4, and SPSS, version 24.

## Results

As the primary measure of this study was introduced into the database in 2007, only subjects with an injury date of 2005 or later were included as they could complete the PART-O measure at the 2nd and 5th year follow-up. Of 12,343 participants with a 2005 injury date or later in the TBIMS-NDB (database as of July 8th, 2019), 11,358 regained command-following before rehabilitation admission and were excluded. After further controlling for DRS motor scores on admission to rehabilitation and number of completed follow-up visits, 333 subjects met inclusion criteria for this study (Figure 1). Demographic and injury characteristic data are presented separately for the RCF (*n* = 229) and nRCF (*n* = 104) groups. The RCF group had a shorter length of stay in acute care (*p* < 0.001). In addition, the RCF group was less disabled on rehabilitation admission and discharge, as measured by the DRS (DRS at admission: *p* < 0.05; DRS at discharge: *p* < 0.001), and more independent at time of rehabilitation discharge (FIM Motor: *p* < 0.001; FIM Cognitive: *p* < 0.001), compared to the nRCF group. Demographic and injury characteristics for the groups determined by presence, or not, of command-following at rehabilitation discharge, are presented in Table 1 and, for the groups defined by days post injury, in Supplementary Table 1.

**Table 1:** Descriptive information for the groups defined by rehab status (Values are quartiles (25th/50th/75th percentiles) or as otherwise Indicated).

	**Overall sample**	**Rehab command-following**	**No rehab command-following**	***p***
n	333	229	104	
Age (Years)	22/30/46	22/30/46	20.25/28.500/46.750	0.345
Years of Education	11.750/12/15	11/12/15	12/12/15	0.332
Male (%)	74.5	76.9	69.2	0.139
Race (%)				0.807
White	65.2	66.4	62.5	
Black	15.0	15.7	13.5	
Asian/Pacific Islander	2.7	2.6	2.9	
Native American	0.9	0.9	1.0	
Hispanic origin	15.0	13.1	19.2	
Other	1.2	1.3	1.0	
GCS Total at ED Admission	3/6/8	3/6/8	3/5/9	0.931
Days spent in acute	20/27/38	19/25/33	25/36/47.250	<0.001
DRS on Admission to Rehab	21/23/24	21/22/24	21/23/25	<0.05
DRS on Discharge from Rehab	8/11/18	7/10/14.500	11/18.500/22.250	<0.001
FIM Motor at Rehab Admission	13/13/14	13/13/14	13/13/13	0.749
FIM Cognitive at Rehab Admission	5/5/5	5/5/5	5/5/5	0.676
FIM Motor at Rehab Discharge	17/40.500/60	27/49/62	13/16/50	<0.001
FIM Cognitive at Rehab Discharge	7/14/19	10/15/20.500	5/7/14.250	<0.001
Days spent in rehab	30/49/79	30/46/73.500	28.750/51.500/91.500	0.441

*p, significance; GCS, Glasgow Coma Scale; ED, Emergency Department; DRS, Disability Rating Scale*.

### Cross-Sectional PART-O Subscale Performance

Cross-sectional analyses comparing PART-O subscale performance at each year showed that, although descriptively the RCF group had higher levels of participation, there were no significant differences in levels of participation between groups in any subscale at any year despite moderate effect sizes (Table 2). Participation scores across subscales and years were notably low with scores ranging from 0.021–1.91 (subscale range: 0–5). Results were similar when groups were assigned based on recovery of command-following within 28 days post-injury (Supplementary Table 2).

**Table 2:** Results of cross-sectional analyses for groups defined by recovery of command-following in rehabilitation (Age, FIM Motor, and FIM Cognitive included as covariates in group comparison) x(sd).

**Subscale**	**Year**	**Overall sample**	**Rehab command-following**	**No rehab command-following**	**Adjusted *p***	**Effect size (Cohen's *d*)**
Productivity	1	0.445 (0.66)	0.564 (0.720) *n* = 201	0.216 (0.440) *n* = 91	0.835	0.583
	2	0.572 (0.762)	0.707 (0.780) *n* = 216	0.305 (0.650) *n* = 94	0.763	0.560
	5	0.704 (0.858)	0.869 (0.880) *n* = 146	0.320 (0.650) *n* = 51	0.315	0.710
Social Relations	1	1.675 (1.047)	1.908 (0.990) *n* = 201	1.206 (1.020) *n* = 91	0.246	0.698
	2	1.699 (1.047)	1.899 (1.030) *n* = 214	1.259 (0.960) *n* = 94	0.222	0.643
	5	1.705 (1.133)	1.909 (1.120) *n* = 146	1.263 (1.030) *n* = 51	0.467	0.600
Out and About	1	1.112 (0.868)	1.310 (0.800) *n* = 201	0.755 (0.871) *n* = 91	0.608	0.664
	2	1.241 (0.871)	1.399 (0.830) *n* = 214	0.912 (0.860) *n* = 94	0.953	0.576
	5	1.438 (0.903)	1.586 (0.870) *n* = 146	1.116 (0.910) *n* = 51	0.879	0.528

*Adjusted p, significance*.

**Figure 2:**
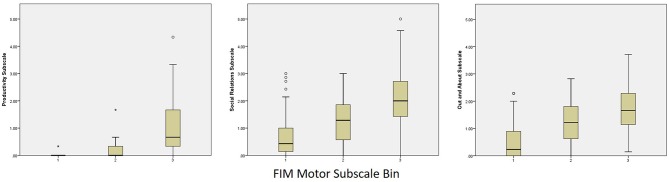
Binned FIM Motor Subscale Performance at Year 5 by PART-O Subscale. FIM bins are as follows: (1) Total Assistance Needed (FIM Motor subscale scores 18–38), (2) Minimal/Moderate Assistance (FIM Motor subscale scores 39–77), (3) Complete Independence (FIM Motor subscale scores 78–91).

**Figure 3:**
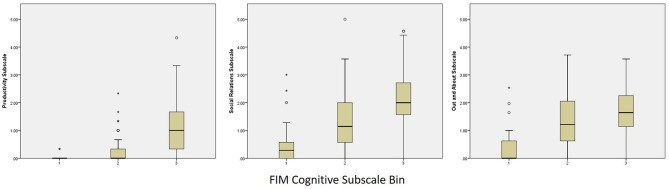
Binned FIM Cognitive Subscale Performance at Year 5 by PART-O Subscale. FIM groups are as follows: (1) Total Assistance Needed (FIM Cognitive subscale scores 5–14), (2) Minimal/Moderate Assistance (FIM Cognitive subscale scores 15–29), (3) Complete Independence (FIM Cognitive subscale scores 30–35).

Categorized performance on the FIM Motor and FIM Cognitive subscales was examined in relation to performance on the PART-O subscales and revealed that participation levels remained low even among subjects who reached ceiling levels of the FIM. In fact, the highest levels of participation across subscales (i.e., scores of 4–5) were attained by fewer than 10 persons across domains and follow-up years. These data were plotted as box and whisker plots in Figures 2, 3 for Motor and Cognitive FIM, respectively.

### Longitudinal PART-O Subscale Performance

Contrary to our hypothesis, there were no between-group longitudinal differences on any of the participation subscales. The FIM Motor subscale was the only variable independently positively associated with participation across subscales.

In the backward elimination model for the PART-O Productivity subscale, both motor and cognitive FIM scores at follow-up predicted level of productivity (*p* < 0.0001 and *p* = 0.0003 respectively, Table 3) with each showing a positive relationship to productivity (estimated partial regression coefficient = β = 0.009 and 0.017, respectively). In addition, there was a negative main effect of age (*p* = 0.0003, β = −0.006) with older individuals having less productivity, as well as a positive main effect of year of follow-up (*p* = 0.004, β = 0.035) indicating that productivity improved over time. The random terms of subject intercept and subject's linear term for year of follow-up were also retained (uncorrelated). All the retained fixed effects in the model accounted for 33% of the variance in PART-O productivity (random and fixed together accounted for 81%).

**Table 3:** Longitudinal mixed-effects model for PART-O Productivity subscale.

**Predictor**	**Unstandardized partial regression coefficient**	**95% CI**	**SE**	***p***
Year	0.035	0.011, 0.058	0.012	0.0036
Age	−0.006	−0.009, −0.003	0.002	0.0003
FIM Motor	0.009	0.006, 0.012	0.002	<0.0001
FIM Cognitive	0.017	0.008, 0.025	0.004	0.0003

*CI, confidence interval; SE, standard error; p, significance*.

Follow-up motor and cognitive FIM scores (*p* < 0.0001 for both, Table 4) predicted Social Relations participation, with each showing a positive relationship to social participation (β = 0.011 and 0.02861, respectively). The random terms of subject intercept and year of follow-up were also retained (correlated *r* = −0.45). The percent variance of social participation accounted for by all of the retained fixed effects in the model was 32.189% (random and fixed accounted for 88%).

**Table 4:** Longitudinal mixed-effects model for PART-O Social Relations subscale.

**Predictor**	**Unstandardized partial regression coefficient**	**95% CI**	**SE**	***p***
FIM Motor	0.011	0.007, 0.015	0.002	<0.0001
FIM Cognitive	0.029	0.016, 0.041	0.006	<0.0001

*CI, confidence interval; SE, standard error; p, significance*.

On the Out and About subscale, higher scores on the follow-up FIM Motor subscale were associated with higher levels of participation (*p* < 0.0001, β = 0.020, Table 5). There was also a main effect of year of follow-up (*p* = 0.0034) and an interaction between age at injury and year of follow-up (*p* = 0.041), whereby the trajectory of increase in follow-up years was sharper for younger participants than for older participants. The random terms of subject intercept and subject's linear term for year of follow-up were also retained (uncorrelated). The percent variance of the PART-O Out and About subscale accounted for by all the retained fixed effects in the model was 42.764% (random and fixed together accounted for 83%).

**Table 5:** Longitudinal mixed-effects model for PART-O Out and About subscale.

**Predictor**	**Unstandardized partial regression coefficient**	**95% CI**	**SE**	***p***
Year	0.086	0.029, 0.144	0.029	<0.005
FIM Motor	0.020	0.018, 0.022	0.001	<0.0001
Age	−0.001	−0.005, 0.004	0.002	0.729
Year by Age interaction	−0.002	−0.003, 0.000	0.001	<0.05

*CI, confidence interval; SE, standard error; p, significance*.

Similarly, in the secondary analyses (when the groups were defined by recovery of command-following by days post injury rather than command-following before or after rehab discharge) there were no significant effects of interest involving group except for an interaction between group and age for the Out and About subscale and a complex three-way interaction of group, age, and linear time for the Productivity subscale (Supplementary Tables 3–5).

For all analyses, residuals from values predicted by the fixed effects as well as from values predicted by the combined fixed and random effects were reasonably normally distributed in accordance with significance test assumptions and indicated good model fit.

## Discussion

We found that persons admitted to inpatient rehabilitation without command-following have profoundly impaired levels of participation years later, even when compared to normative participation levels of individuals currently treated in inpatient rehabilitation (29). Although there was a trend, with moderate effect sizes, in the expected direction of greater participation in the group recovering command-following during rehabilitation, this finding did not reach statistical significance. This result was unexpected, as a substantial proportion of persons who recover command-following during inpatient rehabilitation recover functional independence in the 5 years following injury (5). Notably, consistent with the primary analysis, our secondary analysis of groups defined by whether command-following was recovered before 28 days post injury, did not find a clear effect of group on productivity, or interactions between group and time post injury. However, differences in the results of two analyses, such as a complex 3-way interaction between group, time, and age for the Productivity subscale, suggest more work is needed to better understand the implications of applying varying operational definitions to this patient population. In summary, participation among persons admitted to acute inpatient rehabilitation with traumatic DoC is very low up to 5 years post-injury, even in the subgroup of persons who recover functional independence.

Functional independence was the strongest independent contributor to levels of participation. However, even individuals who regained functional independence demonstrated low levels of participation. This is consistent with prior studies showing that although mobility and participation are related, persons who are independent in this domain have low levels of participation (34). Furthermore, in a randomized control trial of patients with severe TBI undergoing a community rehabilitation program, global function and psychological well-being improved in the experimental group, but participation did not (35). One possible explanation for this unexpected result is that the FIM, and other measures of physical and cognitive function, do not comprehensively measure the complex integration of skills, cognitive processes, physical abilities, and behaviors required to participate in societal and work environments. The high degree of variability in levels of participation among the highest functioning subjects suggests that idiosyncratic qualities of the individual and their environment (e.g., social or personal factors) may be meaningful. A closer examination of non-injury factors (e.g., personality characteristics of caregivers, pre- and post-injury levels of spirituality, etc.) which encourage or inhibit participation may be necessary to improve interventions for this population. Alternatively, an examination of the minority of individuals who do have high levels of functioning and relatively high levels of participation may provide valuable information for intervention development. Future studies are needed to fully determine the quality of the relationship between functioning and participation (i.e., mediator vs. moderator) across the spectrum of injury severity.

The current reimbursement model of health care in the United States focuses on independence in mobility and activities of daily living rather than societal engagement. This focus affects what practitioners prioritize as treatment goals, and therefore may result in failure to address the needs of individuals recovering from a traumatic DoC, especially in post-acute stages. The results of our study suggest that regaining functional independence may be a necessary but insufficient milestone for returning to participatory activities. Rehabilitation aimed at improving participation could be more precisely targeted toward individual's current abilities and the goals deemed valuable for the individual with DoC and their caregiver. For example, clinicians may focus on recovery of basic functions and mobility in some situations and on supported community engagement or independent participation in others. In that latter case, providing access to day-programs, specialized transportation, or supportive social internet sites may be appropriate for higher functioning persons with ongoing cognitive impairments (36, 37). Although still well-below the averages of normative samples, the PART-O Social Relations subscale had higher scores than Productivity and Out and About, suggesting that this domain is the most likely to be modifiable in the setting of impaired cognition or physical function. The effectiveness of cognitive and social therapies aimed at improving participation among persons with a DoC requires further investigation. Furthermore, because participation invariabley involves the interaction between individuals and environment, participation, and its conceptualization, may vary across social supports, cultural values, and healthcare models (38). Substantial global variability in rehabilitation service delivery makes it difficult to quantify the role of rehabilitation in recovery of participation (25), especially at the international level.

Although participation does increase in the 5 years post-injury, this increase is not substantial. This finding may be the result of the constraints of the PART-O as a measurement tool for persons with the most severe injuries. Prior studies reporting functional improvement among persons with a DoC have typically employed the FIM, which was developed to monitor the ability to complete basic activities of daily living in inpatient settings (5, 6). However, the PART-O was developed to measure frequency of engaging in activities in a community setting (4). The behavioral profile of persons with severe brain injury may fall below the floor level of measurement for the PART-O, and therefore, small changes in participation may not be reflected in PART-O scores. For example, an individual may be able to comprehend basic information (as measured by the FIM), while not being able to work for money (as assessed by the PART-O). Furthermore, it is possible that the PART-O does not sufficiently capture the variety of ways that low functioning individuals may participate in society, such as participation in a day program as opposed to being competitively employed or studying toward a degree. Similarly, it is possible that individuals may experience residual motor impairment severe enough to compromise participation without impairing FIM-based activities. Granular measures of participation, that account for what persons with severe TBI and caregivers consider meaningful improvement are needed to develop, apply, and evaluate rehabilitation interventions consistent with the ICF guidelines. Although some persons may not achieve complete reintegration into the workforce or social spheres, a tool that evaluates incremental steps toward these goals is needed.

In interpreting and applying the study findings, several limitations are relevant. Only individuals treated at an acute care hospital and transferred directly to acute inpatient rehabilitation for specialized brain injury care were studied. Clinical services may not be provided in a uniform manner, participants may drop out of the study over time, or inpatient rehabilitation may not be offered for some persons who are not following commands. This may lead to a selection bias or confounding in outcome measures and group assignments. Groups differences in demographic characteristics and data on important pre-injury characteristics (e.g., resilience) were lacking, which may have confounded the analysis (39, 40). Therapy services and medical follow-up in the years after rehabilitation discharge may also differ and were not studied. It is possible that changes to the provision of healthcare services occurred in the data collection window, thus introducing temporal effects (i.e., 2005–2017). However, there have been no major, systematic changes to treatment or access to rehabilitation for persons with impaired consciousness over this time period, making it unlikely that our results were affected by the data collection window. Lastly, self-report and surrogate responses were collapsed for the PART-O. Although few persons with TBI provided self-report PART-O responses, self-report responses may systematically differ from surrogate responses, introducing measurement error, though past work has found that surrogate and patient reports are similar (41). Further, while surrogates may accurately report productive activities and activities outside of the home, given their likely involvement in these activities with the participant, they may not be able to as accurately report on social relationships, which can occur in person, over the phone, or online and may not require surrogate support. We examined participation up to 5 years post-injury. It is likely that participation continues to improve over time, and outcomes at 10 years and beyond should be examined as data become available. We analyzed data from the TBIMS, which provides longitudinal outcome measures across the lifespan of individuals admitted to inpatient rehabilitation facilities in the United States. As a result, our findings may not be applicable outside this system of care. However, in a recent exhaustive report on the global burden of TBI, the issues we identified here (e.g., the need for better approaches to assessing participation, rehabilitation approaches that target the specific needs of patients with DoC, etc) appear to be relevant world-wide (42).

In conclusion, participation remains extremely limited among persons who have experienced a traumatic DoC and are admitted to inpatient rehabilitation with persistent impairments in level of consciousness. This is the case even for persons who regain command-following during rehabilitation and for those who recover functional independence. The incongruity between performance on functional and participatory measures suggests that social or psychological factors as well as the complex integration of behavior, cognition and mobility may be meaningful determinants of levels of participation in the years after severe brain injury.

## Data Availability Statement

The datasets generated for this study can be found in the Traumatic Brain Injury Model Systems National Data and Statistical Center (https://www.tbindsc.org/Researchers.aspx).

## Ethics Statement

The studies involving human participants were reviewed and approved by Partners IRB. The patients/participants provided their written informed consent to participate in this study.

## Author Contributions

YB, KE, CM, JW, JG, FH, and RN-R contributed conception and design of the study. CM organized the database. JL performed the longitudinal statistical analyses. CM, YB, KE, and JL wrote the first draft of the manuscript. All authors contributed to manuscript revision, read and approved the submitted version.

### Conflict of Interest

The authors declare that the research was conducted in the absence of any commercial or financial relationships that could be construed as a potential conflict of interest.
