# Supplementary material for: Participation Following Inpatient Rehabilitation for Traumatic Disorders of Consciousness: A TBI Model Systems Study
Source: Front Neurol. 2019 Dec 18;10:1314. doi: 10.3389/fneur.2019.01314 (PMC6930171; doi:10.3389/fneur.2019.01314)
Supplement: Supplementary file 1 [file Data_Sheet_1.docx]

**Participation Following Inpatient Rehabilitation for Traumatic Disorders of Consciousness: A TBI Model Systems Study**

**Supplementary Material**

**Christopher Malone^1,2^, Kimberly Erler^1,3^, Joseph Giacino^1,2^, Flora M. Hammond^4^, Shannon B. Juengst^5^, Joseph J. Locascio^6^, Risa Nakase-Richardson^7^, Monica Verduzco-Gutierrez^8^, John Whyte^9^, Nathan Zasler^10^, and Yelena Bodien^1,2,6^**

^1^Department of Physical Medicine and Rehabilitation, Harvard Medical School, Boston, Massachusetts, USA

^2^ Neurorehabilitation Laboratory, Spaulding Rehabilitation Hospital, Boston, Massachusetts, USA

^3^ Department of Occupational Therapy, MGH Institute of Health Professions, Boston, Massachusetts, USA

^4^ Department of Physical Medicine and Rehabilitation, Indiana University School of Medicine, Indianapolis, Indiana, USA

^5^ Department of Physical Medicine and Rehabilitation, University of Texas Southwestern Medical Center, Dallas, Texas, USA

^6^ Department of Neurology, Massachusetts General Hospital, Boston, Massachusetts, USA

^7^ Mental Health and Behavioral Science, Defense and Veterans Brain Injury Center, James A. Haley Veterans Hospital; Division of Pulmonary and Sleep Medicine, Department of Internal Medicine, University of South Florida Tampa, Florida, USA

^8^ Department of Physical Medicine and Rehabilitation, McGovern Medical School at the University of Texas Health Science Center at Houston, and TIRR Memorial Hermann, Houston, Texas, USA

^9^ Moss Rehabilitation Research Institute, Elkins Park, Pennsylvania, USA

^10^ Concussion Care Centre of Virginia, Ltd. and Tree of Life Services, Department of Physical Medicine and Rehabilitation, Virginia Commonwealth University, Richmond, Virginia, USA

*** Correspondence:**Corresponding Author: Yelena Bodien
Email: [ybodien@mgh.harvard.edu](mailto:ybodien@mgh.harvard.edu)

**Keywords: traumatic brain injury, minimally conscious state, participation, rehabilitation, outcome.**

| Supplementary Table 1. Descriptive information for the days groups (Values are quartiles (25th/50th/75th percentiles) or as otherwise Indicated) | | | | |
| --- | --- | --- | --- | --- |
|  | Overall Sample | TFC28 | nTFC28 | *p* |
| n | 333 | 54 | 279 |  |
| Age (Years) | 22 / 30 / 46 | 22.250 / 29 / 48.500 | 21 / 30 / 45 | 0.523 |
| Years of Education | 11.750 / 12 / 15 | 11 / 12 / 13.250 | 12 / 12 / 15 | 0.167 |
| Male (%) | 74.5 | 72.2 | 74.9 | 0.678 |
| Race (%) |  |  |  | 0.697 |
| White | 65.2 | 66.7 | 64.9 |  |
| Black | 15.0 | 18.5 | 14.3 |  |
| Asian/Pacific Islander | 2.7 | 3.7 | 2.5 |  |
| Native American | 0.9 | 0 | 1.1 |  |
| Hispanic origin | 15.0 | 9.3 | 16.1 |  |
| Other* | 1.2 | 1.9 | 1.1 |  |
| GCS Total at ED Admission | 3 / 6 / 8 | 4 / 8 / 10.75 | 3 / 5 / 8 | <0.05 |
| Days spent in acute | 20 / 27 / 38 | 14.75 / 18 / 20 | 23 / 31 / 41 | <0.001 |
| DRS on Admission to Rehab | 21 / 23 / 24 | 19.75 / 22 / 23 | 21 / 23 / 24 | <0.05 |
| DRS on Discharge from Rehab | 8 / 11 / 18 | 5.500 / 7.250 / 10 | 8 / 13 / 19 | <0.001 |
| FIM Motor at Rehab Admission | 13 / 13 / 14 | 13 / 13 / 16 | 13 / 13 / 13 | <0.001 |
| FIM Cognitive at Rehab Admission | 5 / 5 / 5 | 5 / 5 / 6.250 | 5 / 5 / 5 | 0.124 |
| FIM Motor at Rehab Discharge | 17 / 40.500 / 60 | 45.750 / 60 / 69.500 | 15 / 35 / 58 | <0.001 |
| FIM Cognitive at Rehab Discharge | 7 / 14 / 19 | 14.75 / 18.500 / 22 | 6 / 12 / 18 | <0.001 |
| Days spent in rehab | 30 / 49 / 79 | 21.75 / 34 / 43 | 32 / 53 / 85 | <0.001 |
| Abbreviations: TFC28 = did regain command-following within 28 days post injury; nTFC28 = did not regain command-following within 28 days post injury; *p* = significance; GCS = Glasgow Coma Scale; ED = Emergency Department; DRS = Disability Rating Scale | | | | |

| Supplementary Table 2. Results of cross-sectional analyses for groups defined by recovery of command-following by days post injury (Age, FIM Motor, and FIM Cognitive included as covariates in group comparison) | | | | | | |
| --- | --- | --- | --- | --- | --- | --- |
| Subscale | Year | Overall Sample | TFC28 x(sd) (n=XXX) | nTFC28 or Longer x(sd) (n=XXX) | *p* | Effect Size (Cohens *d*) |
| Out and About | 1 | 1.112 (0.868) | 1.469 (0.609) n=47 | 1.073 (0.888) n=245 | 0.401 | 0.520 |
|  | 2 | 1.241 (0.871) | 1.636 (0.643) n=49 | 1.177 (0.884) n=259 | 0.868 | 0.594 |
|  | 5 | 1.438 (0.903) | 1.771 (0.825) n=38 | 1.391 (0.908) n=159 | 0.723 | 0.438 |
| Productivity | 1 | 0.445 (0.66) | 0.716 (0.705) n=47 | 0.405 (0.648) n=245 | 0.885 | 0.459 |
|  | 2 | 0.572 (0.762) | 0.986 (0.908) n=49 | 0.510 (0.717) n=261 | 0.164 | 0.582 |
|  | 5 | 0.704 (0.858) | 1.215 (1.008) n=38 | 0.610 (0.781) n=159 | 0.041 | 0.671 |
| Social Relations | 1 | 1.675 (1.047) | 2.078 (0.919) n=47 | 1.615 (1.054) n=245 | 0.578 | 0.468 |
|  | 2 | 1.699 (1.047) | 2.086 (1.035) n=49 | 1.631 (1.041) n=259 | 0.743 | 0.438 |
|  | 5 | 1.705 (1.133) | 2.245 (1.177) n=38 | 1.621 (1.086) n=159 | 0.385 | 0.551 |
| Abbreviations: TFC28 = did regain command-following within 28 days post injury; nTFC28 = did not regain command-following within 28 days post injury; *p* = significance | | | | | | |

| Supplementary Table 3. Longitudinal Mixed-Effects Model for PART-O Out and About Subscale for Groups Defined by Days Post Injury | | | | |
| --- | --- | --- | --- | --- |
| Predictor | Unstandardized Partial Regression Coefficient | 95% CI | SE | *p* |
| Year | 0.087 | 0.030, 0.145 | 0.029 | <0.01 |
| Group Assignment (TFC28) | -0.441 | -0.809, -0.073 | 0.187 | <0.05 |
| FIM Motor | 0.020 | 0.018, 0.022 | 0.001 | <0.001 |
| Age | -0.003 | -0.008, 0.001 | 0.002 | 0.24 |
| Age * Group (TFC28) | 0.013 | 0.004, 0.021 | 0.004 | <0.005 |
| Age*Year | -0.002 | -0.003, -0.0001 | 0.001 | 0.03 |
| Abbreviations: TFC28 = did regain command-following within 28 days post injury; nTFC28 = did not regain command-following within 28 days post injury; CI = confidence interval; SE = standard error; *p* = significance | | | | |

| Supplementary Table 4. Longitudinal Mixed-Effects Model for PART-O Productivity Subscale for Groups Defined by Days Post Injury | | | | |
| --- | --- | --- | --- | --- |
| Predictor* | Unstandardized Partial Regression Coefficient | 95% CI | SE | *p* |
| Year | 0.034 | -0.026, 0.095 | 0.031 | <0.0001 |
| Group (TFC28) | -0.317 | -0.744, 0.110 | 0.217 | 0.145 |
| Age | -0.006 | -0.010, -0.001 | 0.002 | 0.57 |
| FIM Motor | 0.008 | 0.006, 0.011 | 0.001 | <0.0001 |
| FIM Cognitive | 0.016 | 0.007, 0.025 | 0.004 | <0.001 |
| Year * Age * Group(TFC28) | -0.004 | -0.007, -0.0001 | 0.002 | <0.05 |
| Abbreviations: TFC28 = did regain command-following within 28 days post injury; nTFC28 = did not regain command-following within 28 days post injury; CI = confidence interval; SE = standard error; *p* = significance  *For conciseness, two-way interaction terms not shown. | | | | |

| Supplementary Table 5. Longitudinal Mixed-Effects Model for PART-O Social Relations Subscale for Groups Defined by Days Post Injury | | | | |
| --- | --- | --- | --- | --- |
| Predictor | Unstandardized Partial Regression Coefficient | 95% CI | SE | *p* |
| FIM Motor | 0.011 | 0.007, 0.015 | 0.002 | <0.0001 |
| FIM Cognitive | 0.029 | 0.016, 0.041 | 0.006 | <0.0001 |
| Abbreviations: CI = confidence interval; SE = standard error; *p* = significance | | | | |
